# Editorial: MicroRNAs—clinical biomarkers for atrial fibrillation

**DOI:** 10.3389/fcvm.2023.1270461

**Published:** 2023-09-06

**Authors:** Isabel Moscoso, Tania Martins-Marques, Diego Franco

**Affiliations:** ^1^ Cardiology Group, Centre for Research in Molecular Medicine and Chronic Diseases (CIMUS), Universidade de Santiago de Compostela and Department of Cardiology and Coronary Unit and Cellular and Molecular Cardiology Research Unit, Institute of Biomedical Research (IDIS-SERGAS), University Clinical Hospital, Santiago de Compostela, Spain; ^2^ Centro de Investigación Biomédica en Red de Enfermedades Cardiovasculares (CIBERCV), Madrid, Spain; ^3^ Coimbra Institute for Clinical and Biomedical Research (iCBR), Faculty of Medicine, Univ Coimbra, Coimbra, Portugal; ^4^ Center for Innovative Biomedicine and Biotechnology (CIBB), Univ Coimbra, Coimbra, Portugal; ^5^ Clinical Academic Centre of Coimbra (CACC), Coimbra, Portugal; ^6^ Cardiovascular Development Group, Department of Experimental Biology, University of Jaén, Jaén, Spain

**Keywords:** atrial fibrillation, biomarkers, microRNAs, non-coding RNAs, circRNA, meta-analysis, MACE

**Editorial on the Research Topic** MicroRNAs: Clinical biomarkers for atrial fibrillation

Atrial fibrillation (AF) is the most prevalent cardiac arrhythmia in the world, constituting a major risk factor for ischemic stroke (fivefold), death (twofold), and long-term incapacity, with a crucial impact on both health and economic health systems. AF has serious clinical implications on patients’ quality of life, with morbidity from ischemic stroke and heart failure and mortality. Early detection of AF decreases the risk of morbidity and mortality of the patients. Thus, early identification of markers of the disease and response to therapy are valuable to improve disease prevention and patient outcomes.

Small non-coding oligonucleotides, such as microRNAs (miRNAs), are major regulators of gene expression, constituting an important epigenetic mechanism underlying cardiovascular diseases. MiRNAs are expressed in cardiomyocytes, fibroblasts, endothelial cells, and vascular smooth muscle cells, and their regulation has been associated with the pathophysiology of cardiovascular diseases, such as cardiac remodeling and fibrosis, apoptosis, inflammation, proliferation, angiogenesis, and metabolism modulation. Altered expression of miRNAs in the cardiovascular system (both in tissues and circulating) is associated with disorders such as AF, heart failure, atherosclerosis, and other cardiomyopathies. Circulating miRNAs are considered excellent biomarkers in clinical practice. They are obtained with minimal invasive techniques and are secreted by necrotic or living cells. Their profile may be altered in pathophysiological conditions. Moreover, different studies have shown a correlation between miRNA profiles in the cardiac tissue and blood (plasma or serum), opening the possibility of using circulating miRNAs as biomarkers of cardiovascular diseases even in subclinical phases. Using miRNAs as biomarkers may play a crucial role in patient prognosis, namely, by improving risk algorithms in AF, constituting an asset for the management of health system resources.

This Research Topic is focused on the discussion of the use of miRNAs not only as markers of AF diagnosis and prognosis but also as tools for designing novel therapeutic strategies against AF and subsequent development of comorbidities.

An original article by Wei F et al. investigated the potential role of the competitive endogenous RNA-mediated network in AF. Microarray data of circRNA, miRNA, and mRNA from the Gene Expression Omnibus database were used. To select the differentially expressed circRNAs (DECs), these authors used the RobustRankAggreg method, followed by circRNA–miRNA–mRNA-mediated network generation resorting to CircInteractome and miRWalk databases. The resulting circRNA–miRNA–mRNA-mediated network included two circRNAs, four miRNAs, and 83 genes. hsa_circ_0070391 expression levels were found to correlate with left atrial fibrosis in persistent AF and associated with AF prognosis following radiofrequency catheter ablation (Wei et al.). Therefore, this paper advocates in favor of circRNAs as the new biomarkers of atrial fibrosis and AF prognosis.

Atrial fibrosis plays an important role in the onset and development of AF that contribute to structural and electrical conduction abnormalities (1). Non-coding RNAs have been shown to highly correlate with the biological performance of several cardiac cell types and play a pivotal role in the development of cardiac fibrosis. A review by Dong Y et al. aimed to summarize the role of non-coding RNAs in cardiac fibrosis associated with different cardiovascular pathologies, including therein AF, while clarifying the diagnostic and therapeutic potential of non-coding RNAs in cardiac fibrosis (Dong et al.).

A systematic review by Rizal A et al. focused on the study of the correlation between the expression of miRNAs and the development of AF and on the elaboration of the role of genetic factors in the diagnosis of AF. To carry out the bibliographic search, online scientific databases were used, such as Cochrane, ProQuest, PubMed, and Web of Science, using keywords associated with the relationship between miRNAs and AF. The meta-analysis revealed a substantial connection between dysregulation of miR-425-5p expression and AF that showed its potential role as a biomarker (Rizal et al.).

Despite the fact that prophylactic anticoagulation has been shown to have valuable benefits in avoiding comorbidities, patients with AF still experience major adverse cardiovascular events (MACE). Therefore, in recent decades, research focused on the identification of useful biomarkers in risk prevention. A mini review by de los Reyes-García AM et al. summarized the association of specific plasma miRNAs with the development of MACE in AF. At a mechanistic level, miRNAs have been suggested to participate in the development of MACE in AF patients through dysregulation of immunothrombosis. In fact, miRNAs can regulate a formation of neutrophil extracellular traps, which are a key element in the establishment and evolution of thrombotic events (de los Reyes-García et al.). In general, authors detailed the use of miRNAs as both biomarkers and therapeutic targets against thromboinflammatory processes, thereby predicting and preventing the occurrence of MACE in AF.

In conclusion, although the promising results are obtained in a large number of studies, miRNAs are not yet used as biomarkers of AF in clinical practice. This Research Topic highlights the role that miRNA and other non-coding RNAs play and their promising use as diagnostic or therapeutic tools in the management of AF.
